# Molecular evaluation of hepatitis B virus infection and predominant mutations of pre-core, basal core promoter and S regions in an Iranian population with type 2 diabetes mellitus: a case–control study

**DOI:** 10.1186/s12879-022-07528-7

**Published:** 2022-06-17

**Authors:** Fatemeh Farshadpour, Reza Taherkhani, Fatemeh Saberi

**Affiliations:** Department of Virology, School of Medicine, Bushehr University of Medical Sciences, Moallem Street, 751463334 Bushehr, Iran

**Keywords:** Hepatitis B virus, Diabetes mellitus, Prevalence, Genotype, S mutations, Pre-core mutations, Basal core promoter mutations, Iran

## Abstract

**Background:**

This study was designed to evaluate the prevalence, genotypic patterns, and predominant mutations of hepatitis B virus (HBV) infection among diabetic patients.

**Methods:**

Serum samples were obtained from 733 patients with type 2 diabetes mellitus and 782 non-diabetic controls. The presence of HBsAg and HBcAb was determined by ELISA. Nested PCR, targeting S and pre-core regions of the HBV genome, followed by sequencing was carried out to determine HBV genotypes and predominant mutations in the S, basal core promoter (BCP), and pre-core regions of the HBV genome.

**Results:**

Of 733 diabetic patients, 94 cases (12.82%) were positive for HBcAb, 28 cases (3.82%) were positive for HBsAg, and 19 cases (2.59%) had HBV-DNA with genotype D, sub-genotype D1/D3 and subtype ayw2. An occult HBV infection was found in one of the HBV DNA-positive samples, which was positive for HBcAb but negative for HBsAg. P120T/G145R, G1896A/G1899A, and A1762T/G1764T were the most frequent point substitution mutations detected in the S, pre-core, and BCP regions of the HBV genome, respectively. P120T and G145R mutations were associated with low levels or undetectable levels of HBsAg in serum. Therefore, routine tests based on HBsAg detection cannot detect HBsAg-negative infected patients.

**Conclusions:**

Relatively high prevalence of HBV infection was found in diabetic patients, while all of the HBV-infected patients were unaware of their infection. Therefore, screening for HBV infection should be included in the management program of diabetes for timely diagnosis and treatment of infected but asymptomatic patients.

**Supplementary Information:**

The online version contains supplementary material available at 10.1186/s12879-022-07528-7.

## Introduction

Hepatitis B virus (HBV) infection, with approximately 296,000,000 chronic infections globally and an estimated 820,000 deaths annually, is considered to be a challenging public health problem [1]. HBV is a small virus in the family *Hepadnaviridae*, with a partially double-stranded circular DNA genome and enveloped icosahedral capsid. The genome contains four overlapping open reading frames called S, pre-C/*C*, P, and X ORFs, which encode surface proteins (HBsAg), e antigen (HBeAg), and core protein (HBcAg), polymerase, and X protein [2, 3].

The lack of proofreading activity in the replication process of HBV results in the emergence of a high rate of mutations in the HBV genome [4]. The most prevalent mutations that occur naturally during HBV infection are pre-core (pre-C) and basal core promoter (BCP) mutations [5, 6]. The pre-C and BCP mutations favor the persistence of HBV infection and subsequently increase the risk of liver cirrhosis and hepatocellular carcinoma (HCC) [4]. Besides, some mutations in the S region are responsible for the weak response to immunotherapy and HBV vaccination as well as the failure of HBsAg detection [7–9]. Therefore, detection of these mutations can be beneficial in predicting response to HBV vaccination and antiviral treatment as well as the progress of HBV infection in high-risk groups.

Diabetes mellitus, a group of metabolic disorders characterized by hyperglycemia, is recognized as one of the most prevalent non-communicable diseases around the world [10]. There are currently about 422 million diabetic patients and 1.5 million death-related diabetes in the world [11]. Although the cause of diabetes mellitus is not fully understood, several known risk factors, including obesity, aging, chronic inflammation, genetic predisposition, physical inactivity, and urbanization, are thought to promote the development of diabetes mellitus [12]. In addition to the role of genetics, biology and demographic factors, recent studies have highlighted the link between HBV infection and the development of diabetes mellitus [13–16]. The coexistence of HBV infection and diabetes mellitus exhibits a life-threatening condition, which requires immediate consideration. Although nearly 11.9% of the adult population in Iran are diabetics [17], the current knowledge on molecular evaluation of HBV infection among the diabetic population is scarce in Iran. Therefore, this study was designed to evaluate the prevalence, possible risk factors, genotypic patterns, and predominant mutations of HBV infection among patients with type 2 diabetes mellitus.

## Subjects and methods

### Patients and sample collection

All of the patients with type 2 diabetes mellitus attending diabetic clinics of the Bushehr University of Medical Sciences located in southern Iran were included consecutively in this study. In accordance with the American Diabetes Association criteria, diabetic cases were defined as patients with fasting serum glucose levels ≥ 126 mg/dL on at least 2 different occasions accompanied by regular use of oral antidiabetic medications [18]. The demographic, laboratory, and clinical data of each patient were obtained from the patient’s medical records at the diabetic clinics. As a control group, 782 non-diabetic volunteers, who were matched in sex, age (± 3 years), and date of participation with diabetic patients, were recruited from the out-patient populations attending the hospitals of the Bushehr University of Medical Sciences for blood tests. The patients were excluded if they had markers of HCV and/or HIV infections and exhibited evidence of hepatogenous diabetes, gestational diabetes, type 1 diabetes, and autoimmune or metabolic chronic liver disease. All of the participants were informed about the purpose of the research and requested to sign a written informed consent to use their leftover serum samples for HBV detection and analysis. The study was approved by the Ethical Committee of the Bushehr University of Medical Sciences with research project number IR.BPUMS.REC.1395.54 and was funded by the Deputy Research and Affairs of the University with grant number 3254. In addition, all methods were performed under the relevant guidelines and regulations.

### Laboratory diagnosis

All of the serum samples were tested for the detection of hepatitis B surface antigen (HBsAg) and hepatitis B core antibody (HBcAb) using HBsAg one-Version ULTRA and HBc Ab ELISA kits (DIA.PRO, Milan, Italy), respectively. The sensitivity and specificity of these kits were 100%. The HBsAg and/or HBcAb seropositive samples were tested using nested PCR, targeting the S region of the HBV genome, followed by sequencing to determine the genotypic pattern of HBV infection. Briefly, HBV DNA was extracted from serum samples using the High Pure Viral Nucleic Acid kit (Roche, Mannheim, Germany) according to the manufacturer’s instructions. To determine HBV genotypes, 447 nucleotides and 416 nucleotides of the S region were amplified by nested PCR using outer primers [forward primer (244-HBS-F1): GAGTCTAGACTCGTGGTGGACTTC; reverse primer (691-HBS-R1): AAATKGCACTAGTAAACTGAGCCA] and inner primers [forward primer (255-HBS-F2): CGTGGTGGACTTCTCTCAATTTTC; reverse primer (671-HBS-R2): GCCARGAGAAACGGRCTGAGGCCC], respectively [19–21].

To determine mutations in BCP and pre-C regions, the pre-C region was amplified in the first round of PCR using primers 1606-pre-C-F1 (GCATGGAGACCACCGTGAAC) and 2395-pre-C-R1 (AGGCGAGGGAGTTCTTCTTC). The second round of PCR was performed using primers 1653-pre-C-F2 (CATAAGAGGACTCTTGGACT) and 2393-pre-C-R2 (GCGAGGGAGTTCTTCTTC) [21–23]. The 789 bp and 740 bp length fragments from the pre-C region were amplified in the first and second rounds of nested PCR, respectively. The sequences of primers and PCR conditions for amplification of the S and pre-C regions of the HBV genome are summarized in Table 1.

**Table 1:** Sequences of primers for detection of HBV genotypes and mutations

Virus	Primers name	Sequences of Primers 5′ → 3′	Gene	Region in genome	Annealing temperature	Size	References
HBV	244-HBS-F1	GAGTCTAGACTCGTGGTGGACTTC	S	244–267	56 ℃	447 bp	[19–21]
	691-HBS-R1	AAATKGCACTAGTAAACTGAGCCA		668–691			
	255-HBS-F2	CGTGGTGGACTTCTCTCAATTTTC		255–278	56 ℃	416 bp	
	671-HBS-R2	GCCARGAGAAACGGRCTGAGGCCC		648–671			
HBV	1606-pre-C-F1	GCATGGAGACCACCGTGAAC	X and pre-core regions	1606–1625	57 ℃	789 bp	[21–23]
	2395-pre-C-R1	AGGCGAGGGAGTTCTTCTTC		2376–2395			
	1653-pre-C-F2	CATAAGAGGACTCTTGGACT		1653–1672	55 ℃	740 bp	
	2393-pre-C-R2	GCGAGGGAGTTCTTCTTC		2376–2393			

### Genotyping and mutations analyses

The PCR products were analyzed using agarose gel electrophoresis. Following the extraction of amplicons from the agarose gel using the Agarose Gel DNA Extraction Kit (Roche, Mannheim, Germany), the 416 bp length fragments from the S region and the 740 length fragments from the pre-C region were sequenced to determine HBV genotypes and mutations by Sanger dideoxy sequencing technology (Macrogen Co., Korea). The obtained sequences were analyzed by using GenBank Basic Local Alignment Search Tool (BLAST), compared with the reference sequences of S and pre-C regions of the standard HBV genotypes available at the nucleotide database of the NCBI and submitted to the GenBank sequence database. Then, the S and pre-C sequences isolated from the patients and the reference sequences were aligned by the ClustalW program in the Molecular Evolutionary Genetics Analysis (MEGA) software version 7.0 (Biodesign Institute, Tempe, AZ, USA) [24]. The phylogenetic tree was constructed by the neighbor-joining method using MEGA software, as described previously [25]. The reference strains representing the standard HBV genotypes were used as references.

### Statistical analysis

The Student’s *t*-test was used to analyze and compare quantitative variables between HBV-positive and HBV-negative diabetic patients. Categorical variables were compared by *χ*^2^ test or Fisher’s exact test. Logistic regression analysis was used to determine the risk factors associated with the prevalence of HBV infection among diabetic patients, and the odds ratio with 95% confidence intervals was calculated. SPSS 17 package program (SPSS Inc., Chicago, IL, USA) was used for data analyses, and *P* values < 0.05 were considered significant.

## Results

### Baseline demographic

Serum samples were obtained from 733 patients with type 2 diabetes mellitus, including 256 males and 477 females. The mean age ± SD of diabetic patients was 55.2 ± 11.7 years with a range of 26–96 years. The majority of diabetic patients were in the age groups 51–60 years (38.7%) and 61–70 years (20.1%), respectively. Moreover, 782 non-diabetic volunteers, including 304 males and 478 females, were enrolled in the study as the control group. The mean age ± SD of the non-diabetic controls was 53.37 ± 13.36 years with a range of 20–93 years.

### HBV sero-markers and risk factors

Of 733 patients with type 2 diabetes mellitus, 94 cases (12.82%, 95% CI 10.60–15.44%) had HBcAb, and 28 cases (3.82%, 95% CI 2.66–5.47%) were positive for HBsAg. The seroprevalence of HBsAg and HBcAb in the non-diabetic controls were 1.15% (95% CI 0.61–2.17%) and 10.74% (95% CI 8.76–13.11%), respectively. Diabetic patients had a significantly higher seroprevalence of HBsAg than the non-diabetic controls (*P* = 0.01). Although diabetic patients had a higher seroprevalence of HBcAb than the non-diabetic controls, the difference was not statistically significant (*P* = 0.23) (Additional file 1: Table S1).

The highest rate of HBV seroprevalence was observed in the age group 61–70 years (6.1%) for HBsAg and in the age group > 71 years (30.3%) for HBcAb, whereas the lowest HBcAb seroprevalence was found in the age group 26–30 years (6.25%), and those aged < 40 years did not show HBsAg seropositivity (Additional file 1: Tables S2 and S3). Overall, HBV seropositivity increased with age so that HBsAg seropositive diabetic patients had a significantly higher mean age (59.92 ± 13.2) compared to HBsAg seronegative diabetic patients (55.00 ± 11.66) (*P* = 0.03). Besides, HBcAb seropositive diabetic patients showed significantly higher mean age (60.31 ± 12.57) compared to HBcAb seronegative diabetic patients (54.44 ± 11.44) (*P* = 0.001). HBsAg and HBcAb seropositivity were more prevalent in female diabetic patients than male patients, although the differences were not statistically significant (Additional file 1: Tables S2 and S3). HBsAg seropositive diabetic patients had higher AST and ALT levels but lower TCH, TG, and FBS levels than HBsAg seronegative patients. Nevertheless, the seroprevalence of HBsAg among diabetic patients was not statistically associated with the serum levels of liver enzymes, TCH, TG, and FBS (Table 2).

**Table 2:** Comparisons between HBsAg-seropositive and HBsAg-seronegative diabetic patients according to age and biochemical measurements

	Means ± SD of all diabetic participants (733)	Means ± SD of HBsAg negative subjects (705)	Means ± SD of anti- HBsAg positive subjects (28)	*P*-value
Age (year)	55.1937 ± 11.74690	55.0057 ± 11.65605	59.9286 ± 13.20474	0.03
FBS mg/dL	170.5730 ± 72.25192	171.1504 ± 72.59905	156.0357 ± 62.24770	0.28
Chol mg/dL	197.7640 ± 53.02186	198.3730 ± 53.09670	182.4286 ± 49.54566	0.12
TG mg/dL	192.2128 ± 86.48698	193.2610 ± 87.14868	165.8214 ± 63.32693	0.1
ALT IU/L	31.5375 ± 21.55816	31.2979 ± 21.29852	37.5714 ± 27.07114	0.13
AST IU/L	24.8554 ± 13.89916	24.7319 ± 13.90054	27.9643 ± 13.74498	0.23

*FBS* fasting blood sugar; *TG* triglyceride; *ALT* alanine aminotransferase; *AST* aspartate aminotransferase; *HBsAg* hepatitis B surface antigen

HBsAg seroprevalence was higher among the non-diabetic controls aged 70 to 79 years (4.5%), whereas those aged 50–59 years had the lowest rate of HBsAg seroprevalence (0.7%) (Additional file 1: Table S4). HBcAb seroprevalence increased with age so that the non-diabetics over 80 years old had a significantly higher HBcAb seroprevalence (36.7%) compared to the other age groups (*P* = 0.0001) (Additional file 1: Table S5). Besides, HBsAg and HBcAb seroprevalence was more prevalent in male non-diabetics than female non-diabetics, although the differences were not statistically significant (Additional file 1: Tables S4 and S5).

### Molecular evaluation and genotyping

**Fig. 1:**
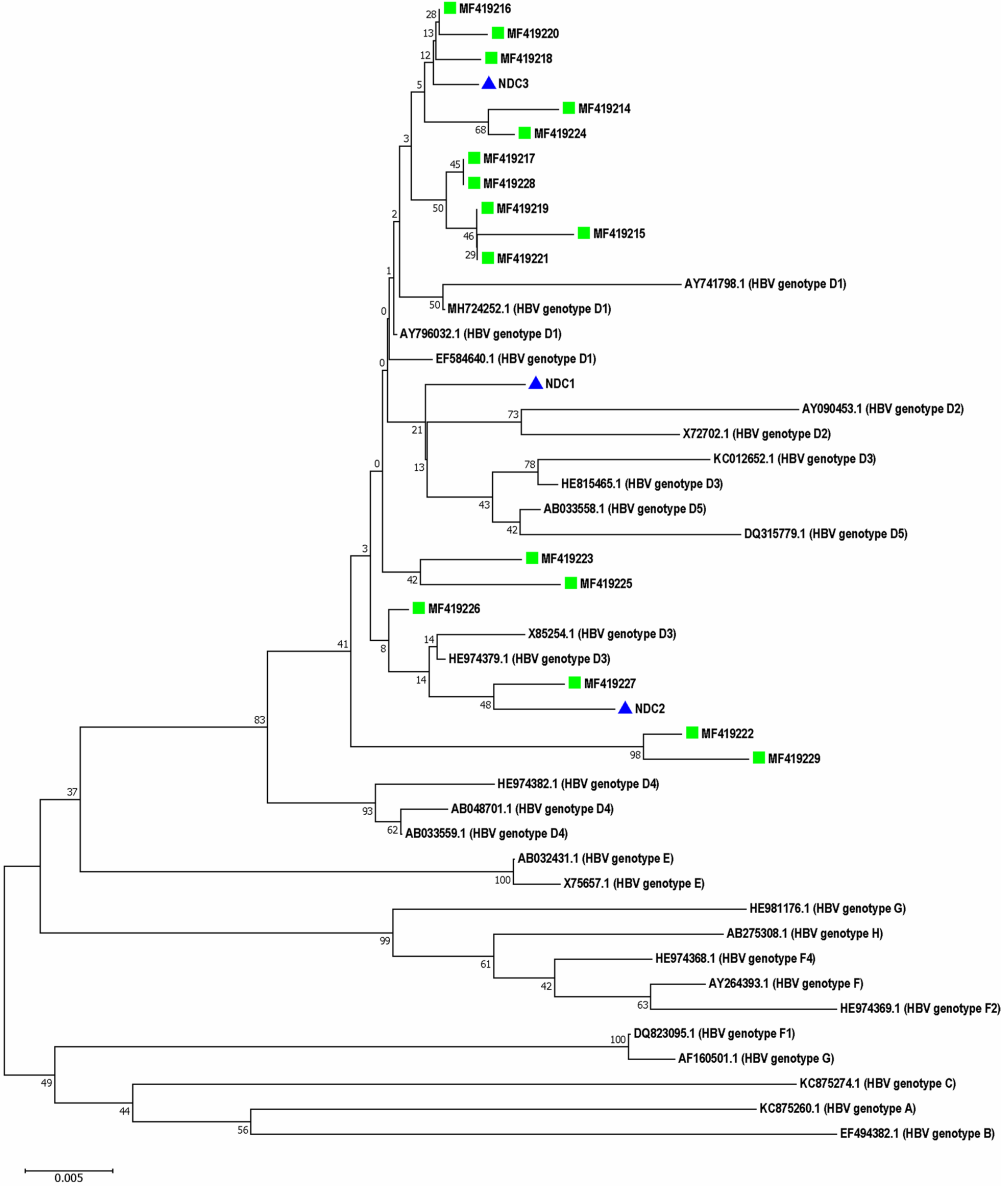
Neighbor-joining phylogenetic tree based on ~ 380 bp nucleotide sequence (~ 270 to 670 bp of the complete reference genome) of the S region of HBV isolates from the serum samples of patients with type 2 diabetes mellitus (green squares) (MF419214–MF419229) and non-diabetic controls (blue triangles) (NDC1–NDC3). Bootstrap resampling strategy and reconstruction were carried out 1000 times to confirm the reliability of the phylogenetic tree

According to the molecular evaluation, 19 diabetic patients (2.59%, 95% CI 1.67–4.01%) had HBV-DNA (Additional file 1: Figs. S1 and S2). The mean age of HBV DNA-positive diabetic patients (60.0 ± 14.3) was higher than that of HBV DNA-negative diabetic patients (55.07 ± 11.66), but this difference was statistically insignificant (*P* = 0.07). Overall, HBV-DNA positivity in diabetic patients was not significantly associated with age, gender distribution, and the serum levels of AST, TCH, TG, and FBS (Table 3). In contrast, HBV-DNA positivity was associated with ALT levels so that those patients with ALT levels of ≥ 81 IU/L (13.0%) had a significantly higher prevalence of HBV-DNA positivity than those patients with ALT levels of ≤ 24 IU/L (1.9%) (OR: 9.60; 95% CI 1.51–61.07; *P* = 0.02). Therefore, an ALT level of ≥ 81 IU/L was the only significant predictive variable for HBV-PCR positivity in diabetic patients (Table 3). An occult HBV infection (OBI) was found in one of the HBV-DNA positive samples, which was positive for HBcAb but negative for HBsAg. The prevalence of HBV-DNA positivity among diabetic patients (2.59%) was significantly higher than HBV-PCR positivity among the non-diabetic controls (0.77%, 95% CI 0.36–1.66%) (*P* = 0.007). None of the non-diabetic controls had occult HBV infection. The HBV sequences isolated from patients with type 2 diabetes mellitus were identified as genotype D, sub-genotype D1/D3, and subtype ayw2 (Fig. 1). The same genotypic pattern was found among the non-diabetic controls. Subtype ayw2 was characterized by Arg (R) at positions 122, Pro (P) at position 127, and Lys (K) at position 160 of HBsAg, but not Phe (F) and Ala (A) at positions 134 and 159 of HBsAg, respectively (Fig. S3**)**. The HBV sequence isolated from diabetic patient with OBI was genotype D, sub-genotype D1/D3, and subtype ayw2.

**Table 3:** Prevalence of HBV-DNA positivity according to demographic and biochemical variables among diabetic patients

	No. of all diabetic participants (%): 733 (100%)	No. of HBV DNA negative subjects (%): 714 (97.4%)	No. of HBV DNA positive subjects (%): 19 (2.6%)	Adjusted OR (95% CI)	*P*-value
Age groups (years)					
26–30	16 (2.2%)	16 (100.0%)	0 (0.0%)	1.0	
31–40	67 (9.1%)	67 (100.0%)	0 (0.0%)	0.00	0.99
41–50	143 (19.5%)	137 (95.8%)	6 (4.2%)	0.00	0.99
51–60	284 (38.7%)	281 (98.9%)	3 (1.1%)	0.24 (0.06–0.99)	0.05
61–70	147 (20.1%)	141 (95.9%)	6 (4.1%)	3.99 (0.98–16.2)	0.05
≥ 71	76 (10.4%)	72 (94.7%)	4 (5.3%)	1.31 (0.36–4.77)	0.69
Gender					
Male	256 (34.9%)	252 (98.4%)	4 (1.6%)	1.0	
Female	477 (65.1%)	462 (96.9%)	15 (3.1%)	2.045 (0.67–6.23)	0.208
FBS (mg/dL)					
≤ 115	128 (17.5%)	124 (96.9%)	4 (3.1%)	1.0	
116–125	89 (12.1%)	87 (97.8%)	2 (2.2%)	0.8 (0.26–2.50)	0.7
≥ 126	516 (70.4%)	503 (97.5%)	13 (2.5%)	1.12 (0.25–5.07)	0.88
Cholesterol (mg/dL)					
≤ 200	403 (55.0%)	391 (97.0%)	12 (3.0%)	0.69 (0.19–2.49)	0.57
201–240	186 (25.4%)	182 (97.8%)	4 (2.2%)	0.97 (0.213–4.39)	0.96
≥ 241	144 (19.6%)	141 (97.9%)	3 (2.1%)	1.0	
TG (mg/dL)					
≤ 150	228 (31.1%)	219 (96.0%)	9 (4.0%)	1.0	
151–200	239 (32.6%)	232 (97.1%)	7 (2.9%)	0.278 (0.1–1.04)	0.06
≥ 201	266 (36.3%)	263 (98.9%)	3 (1.1%)	0.378 (0.1–1.48)	0.16
ALT levels (IU/L)					
≤ 24	324 (44.2%)	318 (98.1%)	6 (1.9%)	1.0	
25–40	256 (34.9%)	248 (96.9%)	8 (3.1%)	1.71 (0.59–4.99)	0.33
41–80	130 (17.7%)	128 (98.5%)	2 (1.5%)	0.48 (0.10–2.31)	0.36
≥ 81	23 (3.1%)	20 (87.0%)	3 (13.0%)	9.60 (1.51–61.07)	0.02
AST levels (IU/L)					
≤ 24	464 (63.3%)	453 (97.6%)	11 (2.4%)	1.0	
25–40	187 (25.5%)	182 (97.3%)	5 (2.7%)	0.88 (0.31–2.58)	0.82
41–80	78 (10.6%)	75 (96.2%)	3 (3.8%)	0.61 (0.34–2.23)	0.45
≥ 81	4 (0.5%)	4 (100.0%)	0 (0.00%)	0.00	0.99

*HBV* hepatitis B virus; *FBS* fasting blood sugar; *TG* triglyceride; *ALT* alanine aminotransferase; *AST* aspartate aminotransferase; *OR* odds ratio; *CI* confidence interval

### Mutation analysis

Frequent point substitution mutations were detected in the S, pre-C, and BCP regions of the HBV genome isolated from the patients. Of those mutations in the S region, the conversion of Pro → Thr at amino acid 120 (P120T) of HBsAg was detected in five samples with HBsAg OD near the cut off of the ELISA kit, and conversion of Gly → Arg at amino acid 145 (G145R) of HBsAg was detected in one sample (Table 4 and Additional file 1: Fig. S3). The S G145R/P120T double mutation was observed in one HBsAg-negative sample. This sample was an OBI case (Accession No. MF419215). In the samples with P120T and/or G145R mutations, HBV-DNA was detectable in the second round of nested PCR. Of those mutations in the pre-C region, a G to A substitution at nucleotide position 1896 (G1896A) was detected in four samples, whereas G1899A mutation was found in eight samples. G1899A mutation was detected in the pre-C region of the occult HBV strain (Accession No. OK382075). Six samples had 5 nucleotide substitutions in the BCP region, including T1753C, A1762T, G1764T, C1766G, and C1773T. These 5 nucleotide substitutions were detected in the BCP region of the occult HBV strain (Accession No. OK382075). There were two samples with pre-C G1896A/G1899A double mutation, while six samples had BCP A1762T/G1764T double mutation. The serum samples with pre-C G1896A mutation exhibited no concomitant BCP A1762T and G1764T mutations; however, six out of the eight samples with pre-C G1899A mutation also exhibited A1762T and G1764T mutations (Table 5 and Additional file 1: Fig. S4). The A1762T and G1764T mutations in the BCP region caused the conversion of Lys → Ile at amino acid 130 (K130I) and Val → Leu at amino acid 131 (V131L) of the X protein (Table 5 and Additional file 1: Fig. S5). I127T, K130I, V131L were detected in the X protein of the occult HBV strain (Accession No. OK382075). The mutation analysis of the OBI case (patient no. 63) has been reported in tables 4 and 5. Moreover, the possible mutations in the S, pre-C, and BCP regions of the HBV genome are shown in Additional file 1: Table S6.

**Table 4:** Characteristics of diabetic patients with point substitution mutations in HBsAg

Accession no.	Patient no.	Sex (M/F)	Age (year)	S mutations	OD of HBsAg ELISA test
MF419214	28	F	69	F93C, M125T, T127P, A168V	3.79
MF419215	63	F	61	**P120T**, M125T, T127P, G130E, **G145R**, A168V	0.09
MF419216	64	M	52	M125T, T127P, A168V	0.138
MF419217	65	F	72	**P120T**, M125T, T127P	0.14
MF419218	66	M	42	M125T, T127P, A168V	0.14
MF419219	69	F	47	**P120T**, M125T, T127P, A168V	0.15
MF419220	71	F	43	L109P, M125T, T127P, A168V	0.166
MF419221	76	F	42	**P120T**, M125T, T127P, A168V	0.221
MF419222	79	F	47	I92T, L94S, V96G, M125T, T127P, G159V	1.425
MF419223	80	F	60	F85C, V96G, M125T, T127P	3.14
MF419224	82	F	96	M125T, T127P, A168V	3.82
MF419225	83	F	66	M125T, T127P	3.4
MF419226	84	F	42	M125T, T127P	3.1
MF419227	85	F	73	M125T, T127P, E164G	2.6
MF419228	86	M	75	**P120T**, M125T	0.246
MF419229	97	F	67	I92T, L94S, V96G, M125T, T127P, G159A, W165R, A168V	1.24

**Table 5:** Characteristics of diabetic patients with point substitution mutations in the pre-C/C, BCP, and X regions of the HBV genome

Accession No	Patient no	Sex (M/F)	Age (year)	OD HBsAg ELISA	Pre-C/C mutation	Pre-C/C nt (1814–2452)	X mutation (1374–1838)	BCP mutation (1742–1849)
OK382075	63	F	61	0.1	G29D, E93D, A109V, N121T, M122A, I145L, P159Q, P164T	G1899A	I127T, K130I, V131L	T1753C, A1762T, G1764T, C1766G, C1773T
OK382076	65	F	72	0.14	G29D, E93D, A109V, N121T, M122A, I145L, P159Q, P164T	G1899A	I127T, K130I, V131L	T1753C, A1762T, G1764T, C1766G, C1773T
OK382077	66	M	42	0.14	G29D, S64A, E93D, A109V, N121T, M122A, I145L, P159Q, P164T	G1899A	I127T, K130I, V131L	T1753C, A1762T, G1764T, C1766G, C1773T
OK382078	69	F	47	0.15	C7R, G29D, E93D, A109V, N121T, M122A, I145L, P159Q, P164T	G1899A	I127T, K130I, V131L	T1753C, A1762T, G1764T, C1766G, C1773T, T1832C
OK382079	71	F	43	0.166	G29D, E93D, A109V, N121T, M122A, I145L, P159Q, P164T	G1899A	I127T, K130I, V131L	T1753C, A1762T, G1764T, C1766G, C1773T
OK382080	80	F	60	3.14	W28*, G29D, S50A, S78T, T96I, A109T, S116G, T120S, I145L, A160P	G1896A, G1899A	I127L	A1727G, A1752C
OK382081	82	F	96	3.82	W28*, S50T, Y67F, E93D, A109V, S116G, N121T, M122V, T143I, P164T	G1896A	I127L	A1752C, A1775C
OK382082	83	F	66	3.4	W28*, C77G, S78T, A109T, A160G	G1896A		T1758C, C1773T, A1775G
OK382083	84	F	42	3.1	E69N, G103V		I127T, K130M, V131I	T1753C, A1762T, G1764A,
OK382084	85	F	73	2.6	V17F, W28*, G29D, S50T, F53Y, S78T, G103A, E106Q, L113Q, I145L, T176C, R180Q, P185S	G1896A, G1899A	I127L	A1752C
OK382085	86	M	75	0.246	G29D, E93D, A109V, N121T, M122A, I145L, P159Q, P164T	G1899A	I127T, K130I, V131L	T1753C, A1762T, G1764T, C1766G, C1773T

## Discussion

In this study, 3.8% and 12.82% of diabetic patients were positive for HBsAg and HBcAb compared to 1.15% and 10.74% of the non-diabetic controls, and 2.59% of diabetic cases had HBV-DNA compared to 0.77% of the non-diabetic controls. Therefore, diabetic patients had a significantly higher prevalence of HBV infection than the controls. Besides, the HBsAg seroprevalence of 3.8% observed in diabetic patients is considerably higher than the HBsAg prevalence of 0.15% reported in the blood donors of this region [26]. Moreover, the HBV prevalence reported in this study is higher than the overall prevalence of HBV in the general population of Iran [27]. The high prevalence of HBV infection in diabetic cases might be due to the possible role of HBV in the development of diabetes mellitus through induction of insulin resistance associated with persistent inflammatory reactions in response to HBV infection and overproduction of tumor necrosis factor-α and nitric oxide in the liver which are involved in the destruction of the insulin metabolic action, destruction of β-cell of the islet due to the replication of HBV in the pancreas, or induction of glycometabolism disorders due to hepatic damage caused by HBV infection [14, 15]. On the other hand, the risk factors associated with diabetic patients, including frequent blood glucose monitoring, hospitalization, and medical interventions, increase the risk of exposure to HBV [16]. In addition to parenteral transmission of HBV, defective cellular immune responses due to altered levels of T-lymphocyte subsets increase the risk of infection in diabetic patients [16]. Furthermore, concurrent diabetes mellitus increases the risk of hepatocarcinogenesis in HBV-infected patients [28, 29].

The association between hepatitis B and diabetes mellitus remains controversial. Some studies demonstrated an association between HBV infection and diabetes mellitus [13–16], while no such association was reported in some other studies [10, 30]. These studies were performed in different geographical regions with different sample sizes and failed to reach a consensus. The current study indicates that diabetic patients have a higher risk of HBV infection when compared with non-diabetic subjects; however, given the fact that this is a cross-sectional study, prospective cohort studies are required to confirm this assertion and to achieve further understanding of the possible link between HBV infection and diabetes mellitus in an Iranian population.

The seroprevalence of 3.8% for HBsAg reported in this study is higher than those observed among diabetic patients in Italy (1.63%) [31], the Kurdistan Region of Iraq (2.13%) [32], and Ethiopia (3.7%) [33] but lower than those reported among diabetic patients in China (13.5%) [34] and Taiwan (13.54%) [35]. Moreover, the seroprevalence of 12.82% for HBcAb observed in this study is higher than that reported among diabetic patients in the United States (8.2%) [36] but lower than those reported among diabetic patients in Brazil (16.8%) [37] and China (62.3%) [34]. Differences in immunization status, risk factors, the burden of HBV infection in the general population, the levels of safety measures in public health centers, preventive strategies, and risk of exposure to HBV in different regions as well as differences in the sensitivity and specificity of diagnostic methods, study period, sociodemographic characteristics of the study population and number of participants in different studies might explain these variations in the seroprevalence of HBV infection in different parts of the world.

In the present study, HBsAg seropositivity increased with age, from 3% in the age group 41–60 years to 6.7% in the age group over 60 years, while all diabetic patients under 40 years old were negative for HBsAg. This is probably due to the implementation of the HBV vaccination program for infants in 1993 and the vaccination of teenagers since 2006 in Iran [26]. Our findings are in accordance with the results of a previous study among blood donors in the South of Iran, which has demonstrated a significant association between older ages and higher HBsAg seroprevalence [26]. Besides, all of the HBV-infected diabetic patients were unaware of their infection, although they had higher AST and ALT levels than uninfected diabetic patients. A study from Italy also reported higher levels of liver enzymes in infected diabetic patients compared to uninfected cases [31]. In the clinic, elevated liver enzymes are usually considered signs of metabolic disease, not signs of HBV infection.

In this study, P120T/G145R, G1896A/G1899A, and A1762T/G1764T were the most frequent point substitution mutations detected in the S, pre-core, and BCP regions of HBV isolated from the patients, respectively. P120T and G145R mutations in the S region were associated with low levels or undetectable levels of HBsAg in serum. Therefore, routine tests based on HBsAg detection cannot detect HBsAg-negative infected patients. Besides, HBV with P120T and G145R mutations is thought to be a vaccine-induced immune escape mutant [9], since the patient with these two mutations has been immunized with the HBV vaccine. A1762T and G1764T mutations in the BCP region were detected in six samples. A high prevalence of A1762T and G1764T mutations has been reported in patients with liver fibrosis, cirrhosis, and HCC [4, 38]. The G1896A and G1899A mutations in the pre-C region were detected in four and eight samples, respectively. The pre-C mutations have a significant association with remission of liver disease [5]. Considering the critical impact of these mutations on the clinical course and treatment of HBV infection, prompt detection of these mutants in patients with chronic HBV infection is important and can improve the diagnostics, vaccination, and treatment strategies. According to the current HBV treatment strategies, tenofovir and entecavir are used in the treatment of chronic hepatitis B in Iran [39]. Deletion, insertion, or frameshifting mutations were not observed in our isolates.

Based on the nucleotide sequence analysis of the S region, HBV genotype D with sub-genotype D1/D3, and subtype ayw2 were found in our HBV DNA-positive diabetic patients. The same genotypic pattern has been reported in a previous study from southern Iran [40]. Genotype D with nine sub-genotypes (D1–D9) shows a widespread distribution and is predominant in the Mediterranean region, Europe, Africa, India, and Indonesia [41]. This genotype is characterized by chronicity, progression to cirrhosis and HCC, mutation frequency, and a low response to interferon-based therapy [41, 42]. HBV has been classified into 4 major serotypes (ayr, adw, adr and ayw) and 10 related subtypes (ayr, adw2, adw3, adw4, adrq+, adrq−, ayw1, ayw2, ayw3 and ayw4) according to the variations in amino acids at positions 122 and 160 [43]. Moreover, the frequency of mutations has a significant association with the predominant genotypic pattern of HBV in distinct geographical locations. For example, a high frequency of A1762T and G1764T mutations in the BCP region are associated with HBV genotypes C and D [7]. Besides, the pre-C G1896A mutation is characteristic of HBV genotypes B, D, and E [5], and subtype ayw is associated with genotypes D and E [2].

This is the first report on the molecular evaluation and mutation analysis of HBV infection among diabetic patients in Iran. However, the cross-sectional design of the study prevents follow-up of the infected patients. Therefore, the possible effects of these mutations on the disease progression remained unclear among diabetic patients. As another limitation, this study was not able to determine the relationship between HBV infection and diabetes mellitus. Therefore, whether HBV infection triggers the onset of diabetes or diabetes increases the risk of HBV infection remains to be determined by prospective or longitudinal studies.

## Conclusion

The present study indicates a relatively high prevalence of HBV infection among patients with type 2 diabetes mellitus, which may remain undiagnosed in the absence of testing. Considering the complications associated with the coexistence of hepatitis B and diabetes, vaccination and screening of all diabetic patients for HBV infection as well as timely treatment of infected patients are recommended to reduce the incidence and risk of HBV infection among the diabetic population. Moreover, we observed P120T and G145R mutations in the S region, which were associated with low levels or undetectable levels of HBsAg in serum or plasma. Therefore, routine tests based on HBsAg detection cannot detect HBsAg-negative infected patients. Besides, A1762T/G1764T mutations in the BCP region and G1896A/G1899A mutations in the pre-C region were detected in our HBV-infected diabetic patients. Detection of these mutations can provide information regarding disease progression in patients with chronic HBV infection.

## Supplementary Information

**Additional file 1: Table S1.** Prevalence of HbsAg, HBcAb and HBV viremia in diabetics patients and non-diabetic controls. **Table S2.** Prevalence of HBsAg according to demographic and biochemical variables among diabetic patients. **Table S3.** Prevalence of HBcAb according to demographic characteristics among diabetic patients. **Table S4.** Prevalence of HBsAg according to demographic characteristics among non-diabetic controls. **Table S5.** Prevalence of HBcAb according to demographic characteristics among non-diabetic controls. **Table S6.** Mutations in HBV genome. **Figure S1.** The PCR amplification of the S region of HBV genome extracted from the serum samples of diabetic patients. L, 100-bp DNA ladder; N, negative control; P, positive control; 2-7, 9-11, 14 and 19, amplified product (≈417 bp) on 2% agarose gel electrophoresis. **Figure S2.** The PCR amplification of the X and pre-core regions of HBV genome extracted from the serum samples of diabetic patients. L, 100-bp DNA ladder; N, negative control; P, positive control; 2–7, amplified product (≈735 bp) on 2% agarose gel electrophoresis. **Figure S3.** Alignment of the amino acid sequences of HBsAg (64 aa to 173 aa) of strains isolated from the diabetic patients (GenBank accession Nos. MF419214–MF419229) and the reference sequences available at the nucleotide database of the NCBI. **Figure S4.** Alignment of amino acid sequences of the pre-core region isolated from the diabetic patients (GenBank accession Nos. OK382075-OK382085) and the reference sequences available at the nucleotide database of the NCBI. **Figure S5.** Alignment of 109 to 154 amino acid sequences of the X protein (1698 to 1838 nucleotide sequence) of strains isolated from the diabetic patients (GenBank accession Nos. OK382075-OK382085) and the reference sequences available at the nucleotide database of the NCBI.

## Data Availability

All relevant data are within the paper or supplementary material. The S, pre-core, and X sequences isolated from diabetic patients have been submitted to the GenBank sequence database under GenBank accession Nos. MF419214–MF419229, OK382075-OK382085 and OK382075-OK382085, respectively. https://www.ncbi.nlm.nih.gov/popset?DbFrom=nuccore&Cmd=Link&LinkName=nuccore_popset&IdsFromResult=1391900023.
