# Supplementary material for: Molecular evaluation of hepatitis B virus infection and predominant mutations of pre-core, basal core promoter and S regions in an Iranian population with type 2 diabetes mellitus: a case–control study
Source: BMC Infect Dis. 2022 Jun 17;22:553. doi: 10.1186/s12879-022-07528-7 (PMC9206294; doi:10.1186/s12879-022-07528-7)
Supplement: Supplementary file 1 — Additional file 1: Table S1. Prevalence of HbsAg, HBcAb and HBV viremia in diabetics patients and non-diabetic controls. Table S2. Prevalence of HBsAg according to demographic and biochemical variables among diabetic patients. Table S3. Prevalence of HBcAb according to demographic characteristics among diabetic patients. Table S4. Prevalence of HBsAg according to demographic characteristics among non-diabetic controls. Table S5. Prevalence of HBcAb according to demographic characteristics among non-diabetic controls. Table S6. Mutations in HBV genome. Figure S1. The PCR amplification of the S region of HBV genome extracted from the serum samples of diabetic patients. L, 100-bp DNA ladder; N, negative control; P, positive control; 2-7, 9-11, 14 and 19, amplified product (≈417 bp) on 2% agarose gel electrophoresis. Figure S2. The PCR amplification of the X and pre-core regions of HBV genome extracted from the serum samples of diabetic patients. L, 100-bp DNA ladder; N, negative control; P, positive control; 2–7, amplified product (≈735 bp) on 2% agarose gel electrophoresis. Figure S3. Alignment of the amino acid sequences of HBsAg (64 aa to 173 aa) of strains isolated from the diabetic patients (GenBank accession Nos. MF419214–MF419229) and the reference sequences available at the nucleotide database of the NCBI. Figure S4. Alignment of amino acid sequences of the pre-core region isolated from the diabetic patients (GenBank accession Nos. OK382075-OK382085) and the reference sequences available at the nucleotide database of the NCBI. Figure S5. Alignment of 109 to 154 amino acid sequences of the X protein (1698 to 1838 nucleotide sequence) of strains isolated from the diabetic patients (GenBank accession Nos. OK382075-OK382085) and the reference sequences available at the nucleotide database of the NCBI. [file 12879_2022_7528_MOESM1_ESM.doc]

**Table S1.** Prevalence of HbsAg, HBcAb and HBV viremia in diabetics patients and non-diabetic controls

|  | **No. of all non-diabetic participants (%): 782 (100%)** | **No. of all diabetic participants (%): 733 (100%)** | ***P*-Value** |
| --- | --- | --- | --- |
| **HBsAg** |  |  | **0.01** |
| Negative | 773 (98.85%) | 705 (96.18%) |  |
| Positive | 9 (1.15%) | 28 (3.82%) |  |
| **HBcAb** |  |  | **0.23** |
| Negative | 698 (89.26%) | 639 (87.18%) |  |
| Positive | 84 (10.74%) | 94 (12.82%) |  |
| **HBV DNA** |  |  | **0.007** |
| Negative | 776 (99.23%) | 714 (97.41%) |  |
| Positive | 6 (0.77%) | 19 (2.59%) |  |

**Table S2.** Prevalence of HBsAg according to demographic and biochemical variables among diabetic patients

|  | **No. of all diabetic participants (%): 733 (100%)** | **No. of HBsAg negative subjects (%): 705 (96.2%)** | **No. of HBsAg positive subjects (%): 28 (3.8%)** | **Adjusted OR**  **(95% CI)** | ***P*-Value** |
| --- | --- | --- | --- | --- | --- |
| **Age groups (years)** |  |  |  |  |  |
| 26-30 | 16 (2.2%) | 16 (100.0%) | 0 (0.0%) | 1.0 |  |
| 31-40 | 67 (9.1%) | 67 (100.0%) | 0 (0.0%) | 0.00 | 0.99 |
| 41-50 | 143 (19.5%) | 135 (94.4%) | 8 (5.6%) | 0.00 | 0.99 |
| 51-60 | 284 (38.7%) | 279 (98.2%) | 5 (1.8%) | 0.3 (0.1-0.94) | 0.04 |
| 61-70 | 147 (20.1%) | 138 (93.9%) | 9 (6.1%) | 3.64 (1.2-11.1) | 0.02 |
| ≥ 71 | 76 (10.4%) | 70 (92.1%) | 6 (7.9%) | 1.31 (0.45-3.84) | 0.62 |
| **Gender** |  |  |  |  |  |
| Male | 256 (34.9%) | 251 (98.0%) | 5 (2.0%) | 1.0 |  |
| Female | 477 (65.1%) | 454 (95.2%) | 23 (4.8%) | 2.54 (0.95-6.77) | 0.06 |
| **FBS (mg/dL)** |  |  |  |  |  |
| ≤ 115 | 128 (17.5%) | 124 (96.9%) | 4 (3.1%) | 1.0 |  |
| 116–125 | 89 (12.1%) | 85 (95.5%) | 4 (4.5%) | 1.46 (0.36-5.99) | 0.6 |
| ≥ 126 | 516 (70.4%) | 496 (96.1%) | 20 (3.9%) | 0.86 (0.29-2.57) | 0.78 |
| **TCH (mg/dL)** |  |  |  |  |  |
| ≤ 200 | 403 (55.0%) | 383 (95.0%) | 20 (5.0%) | 1.0 |  |
| 201–240 | 186 (25.4%) | 182 (97.8%) | 4 (2.2%) | 2.38 (0.8-7.05) | 0.12 |
| ≥ 241 | 144 (19.6%) | 140 (97.2%) | 4 (2.8%) | 1.83 (0.61-5.44) | 0.28 |
| **TG (mg/dL)** |  |  |  |  |  |
| ≤ 150 | 228 (31.1%) | 217 (95.2%) | 11 (4.8%) | 1.0 |  |
| 151–200 | 239 (32.6%) | 229 (95.8%) | 10 (4.2%) | 1.16 (0.48-2.79) | 0.74 |
| ≥ 201 | 266 (36.3%) | 259 (97.4%) | 7 (2.6%) | 1.88 (0.72-4.92) | 0.2 |
| **ALT levels (IU/L)** |  |  |  |  |  |
| ≤ 24 | 324 (44.2%) | 316 (97.5%) | 8 (2.5%) | 1.0 |  |
| 25–40 | 256 (34.9%) | 244 (95.3%) | 12 (4.7%) | 1.94 (0.78-4.83) | 0.15 |
| 41–80 | 130 (17.7%) | 125 (96.2%) | 5 (3.8%) | 0.81 (0.28-2.36) | 0.7 |
| ≥ 81 | 23 (3.1%) | 20 (87.0%) | 3 (13.0%) | 3.75 (0.83-16.93) | 0.09 |
| **AST levels (IU/L)** |  |  |  |  |  |
| ≤ 24 | 464 (63.3%) | 447 (96.3%) | 17 (3.7%) | 1.0 |  |
| 25–40 | 187 (25.5%) | 180 (96.3%) | 7 (3.7%) | 1.02 (0.42-2.51) | 0.96 |
| 41–80 | 78 (10.6%) | 74 (94.9%) | 4 (5.1%) | 1.4 (0.4-4.9) | 0.6 |
| ≥ 81 | 4 (0.5%) | 4 (100.0%) | 0 (0.00%) | 0.00 | 0.99 |

HBsAg, Hepatitis B surface antigen; FBS, fasting blood sugar; TCH, total cholesterol; TG, triglyceride; ALT, alanine transaminase; AST, aspartate transaminase; OR, odds ratio; CI, confidence interval

**Table S3.** Prevalence of HBcAb according to demographic characteristics among diabetic patients

|  | **No. of all diabetic participants (%): 733 (100%)** | **No. of HBcAb negative subjects (%): 639 (87.18%)** | **No. of HBcAb positive subjects (%): 94 (12.82%)** | **Adjusted OR**  **(95% CI)** | ***P*-Value** |
| --- | --- | --- | --- | --- | --- |
| **Age groups (years)** |  |  |  |  |  |
| 26-30 | 16 (2.2%) | 15 (93.75%) | 1 (6.25%) | 1.0 |  |
| 31-40 | 67 (9.1%) | 60 (89.5%) | 7 (10.5%) | 1.750 (0.2-15.33) | 0.61 |
| 41-50 | 143 (19.5%) | 132 (92.3%) | 11 (7.7%) | 0.714 (0.26-1.93) | 0.51 |
| 51-60 | 284 (38.7%) | 256 (90.1%) | 28 (9.9%) | 1.312 (0.63-2.72) | 0.46 |
| 61-70 | 147 (20.1%) | 123 (83.7%) | 24 (16.3%) | 1.784 (0.99-3.21) | 0.05 |
| >71 | 76 (10.4%) | 53 (69.7%) | 23 (30.3%) | 2.22 (1.15-4.29) | 0.02 |
| **Gender** |  |  |  |  |  |
| Male | 256 (34.9%) | 224 (87.5%) | 32 (12.5%) | 1.0 |  |
| Female | 477 (65.1%) | 415 (87.0%) | 62 (13.0%) | 1.05 (0.66-1.65) | 0.85 |

HBcAb, Hepatitis B core antibody; OR, odds ratio; CI, confidence interval

**Table S4.** Prevalence of HBsAg according to demographic characteristics among non-diabetic controls

|  | **No. of all non-diabetic participants (%): 782 (100%)** | **No. of HBsAg negative subjects (%):773 (98.85%)** | **No. of HBsAg positive subjects (%):9 (1.15%)** | ***P*-Value** |
| --- | --- | --- | --- | --- |
| **Age groups (years)** |  |  |  | **0.2** |
| ≤39 | 90 (11.5%) | 90 (100.0%) |  |  |
| 40-49 | 174 (22.3%) | 172 (98.9%) | 2 (1.1%) |  |
| 50-59 | 288 (36.8%) | 286 (99.3%) | 2 (0.7%) |  |
| 60-69 | 156 (19.9%) | 154 (98.7%) | 2 (1.3%) |  |
| 70-79 | 44 (5.6%) | 42 (95.5%) | 2 (4.5%) |  |
| ≥80 | 30 (3.8%) | 29 (96.7%) | 1 (3.3%) |  |
| **Gender** |  |  |  | **0.489** |
| Female | 478 (61.1%) | 471 (99%) | 5 (1%) |  |
| Male | 304 (38.9%) | 300(98.7%) | 4 (1.3%) |  |

**Table S5.** Prevalence of HBcAb according to demographic characteristics among non-diabetic controls

|  | **No. of all non-diabetic participants (%): 782 (100%)** | **No. of HBcAb negative subjects (%):698 (89.3%)** | **No. of HBcAb positive subjects (%):84 (10.7%)** | ***P*-Value** |
| --- | --- | --- | --- | --- |
| **Age groups (years)** |  |  |  | **0.0001** |
| ≤39 | 90 (11.5%) | 82 (91.1%) | 8 (8.9%) |  |
| 40-49 | 174 (22.3%) | 159 (91.4%) | 15 (8.6%) |  |
| 50-59 | 288 (36.8%) | 264 (91.7%) | 24 (8.3%) |  |
| 60-69 | 156 (19.9%) | 141 (90.4%) | 15 (9.6%) |  |
| 70-79 | 44 (5.6%) | 33 (75.0%) | 11 (25.0%) |  |
| ≥80 | 30 (3.8%) | 19 (63.3%) | 11 (36.7%) |  |
| **Gender** |  |  |  | **0.126** |
| Female | 478 (61.1%) | 432 (90.4 %) | 46 (9.6%) |  |
| Male | 304 (38.9%) | 266 (87.5%) | 38 (12.5%) |  |

**Table S6.** Mutations in HBV genome

| **Region** | **Mutations** |
| --- | --- |
| **S region/HBsAg** | C48G, F85C, V96A, I92T, F93C, L94S, V96G, M103I, L109I/P, T116N, T118K, P120A/E/T/S, K122R/I, T123N, M125T, I/T126A/N/I/S, T127P, Q129H/R, G130E, M133L, L134S, Y134H, K141E, P142S, S143L, D144A/E/V, G145R/A, G159A/V, E164G, W165R, A168V, S171F, L175S, G185E and V190A  Insertion at aa 121-124 |
| **Pre-C/C region** | G1862T, G1896A and G1899A  Start-codon mutations at positions 1814 or 1815  Nonsense mutation at position 1874  Frame shift mutations  Stop-codon mutations at positions 1817–1819 (CAA to TAA), 1874–1876 (AAG to TAG) and 1895–1897 (TGG to TAG, TGA or TAA)  C7R, V17F, W28*, G29D, S50A/T, F53Y, S64A, Y67F, E69N, C77G, S78T, E93D, T96I, G103A/V, E106Q, A109T/V, L113Q, S116G, T120S, N121T, M122A/V, T143I, I145L, P159Q, A160G/P, P164T, T176C, R180Q and P185S |
| **Basal core promoter (BCP)** | A1727G, A1752C/G, T1753C, T1758C, A1762T, G1764T/A, C1766G/T, C1773T, A1775C/G, T1832C, A1846T, G1896A and G1899A  Deletion of nt. 1758-1765 or 1768-1775 |
| **X protein** | I127L/T, K130I/M and V131L/I |


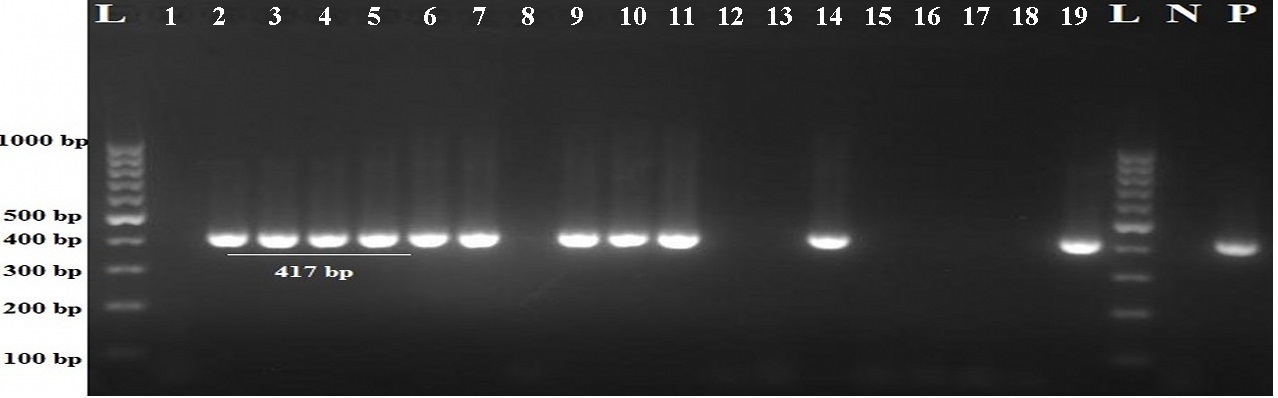


**Fig. S1.** The PCR amplification of the S region of HBV genome extracted from the serum samples of diabetic patients. L, 100-bp DNA ladder; N, negative control; P, positive control; 2-7, 9-11, 14 and 19, amplified product (≈417 bp) on 2% agarose gel electrophoresis


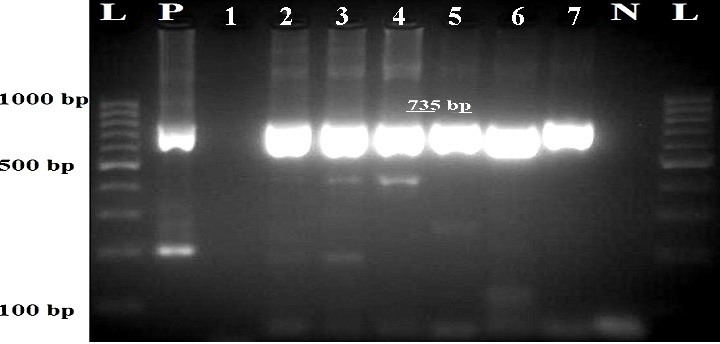


**Fig. S2.** The PCR amplification of the X and pre-core regions of HBV genome extracted from the serum samples of diabetic patients. L, 100-bp DNA ladder; N, negative control; P, positive control; 2–7, amplified product (≈735 bp) on 2% agarose gel electrophoresis


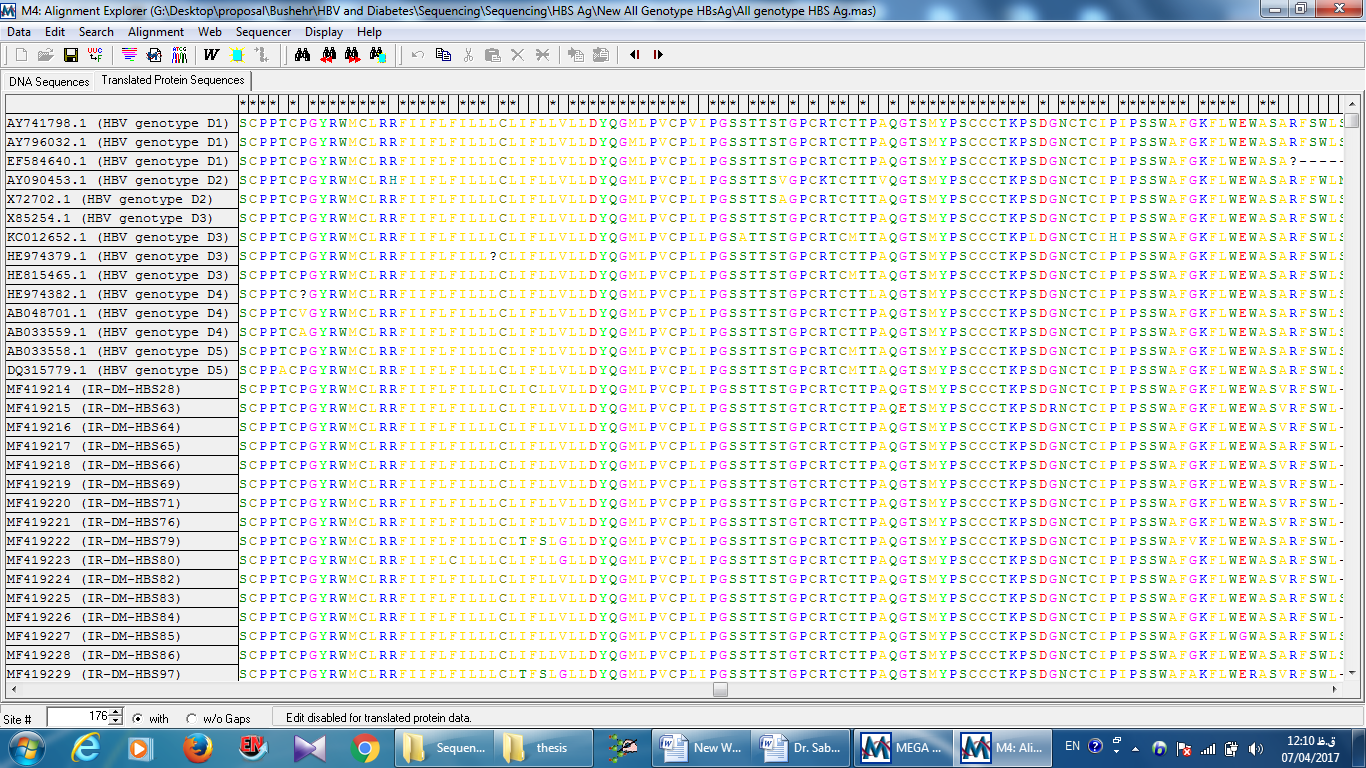


**Fig. S3.** Alignment of the amino acid sequences of HBsAg (64 aa to 173 aa) of strains isolated from the diabetic patients (GenBank accession Nos. MF419214–MF419229) and the reference sequences available at the nucleotide database of the NCBI


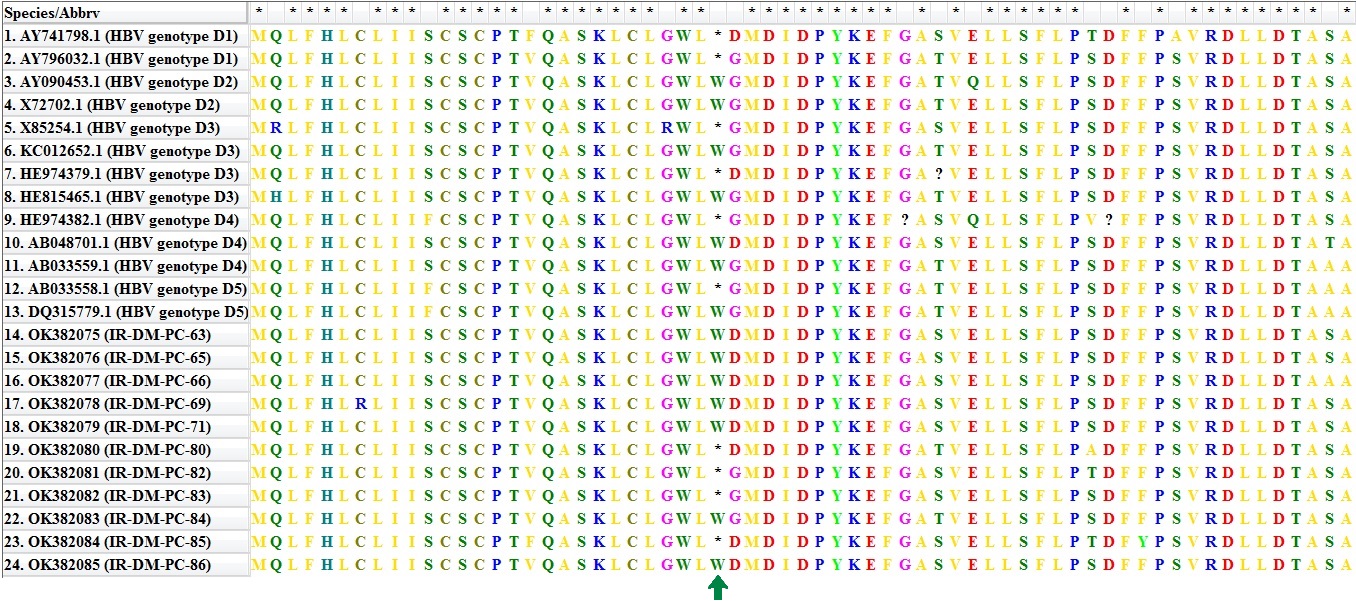


**Fig. S4.** Alignment of amino acid sequences of the pre-core region isolated from the diabetic patients (GenBank accession Nos. OK382075-OK382085) and the reference sequences available at the nucleotide database of the NCBI


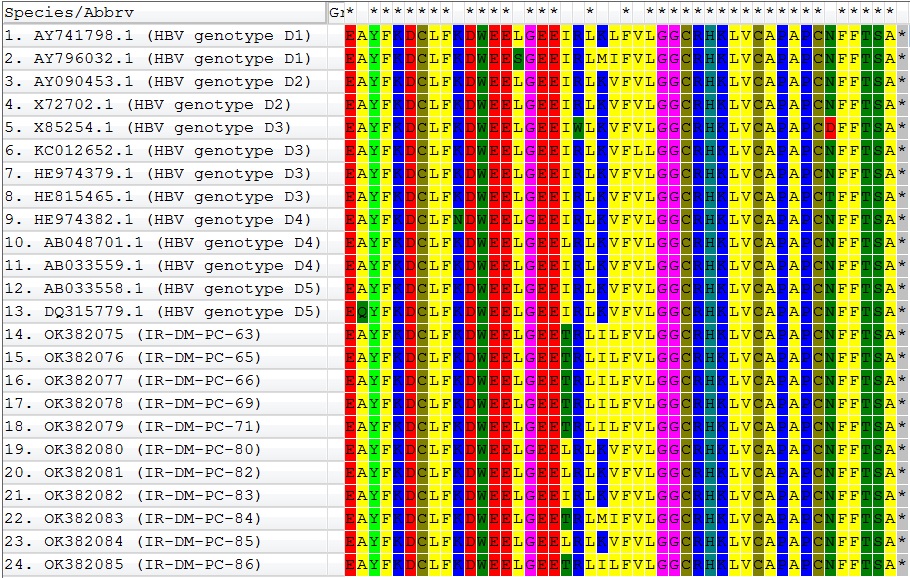


**Fig. S5.** Alignment of 109 to 154 amino acid sequences of the X protein (1698 to 1838 nucleotide sequence) of strains isolated from the diabetic patients (GenBank accession Nos. OK382075-OK382085) and the reference sequences available at the nucleotide database of the NCBI


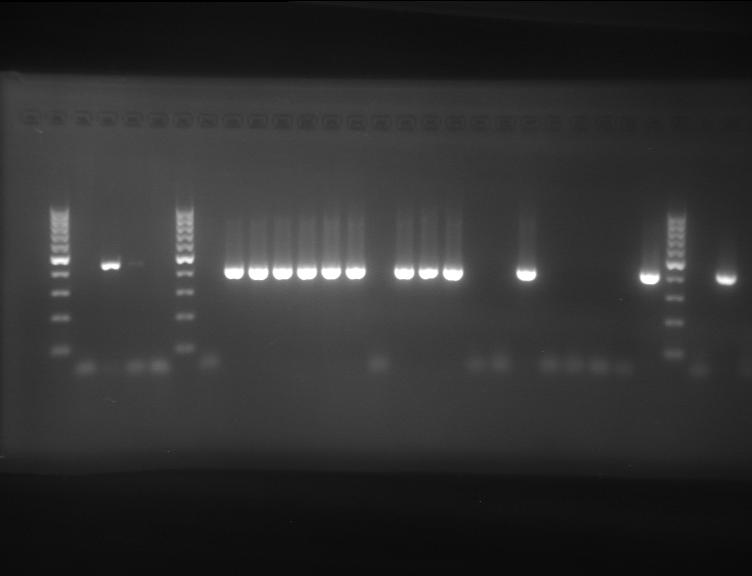


**Fig. S1 Original**


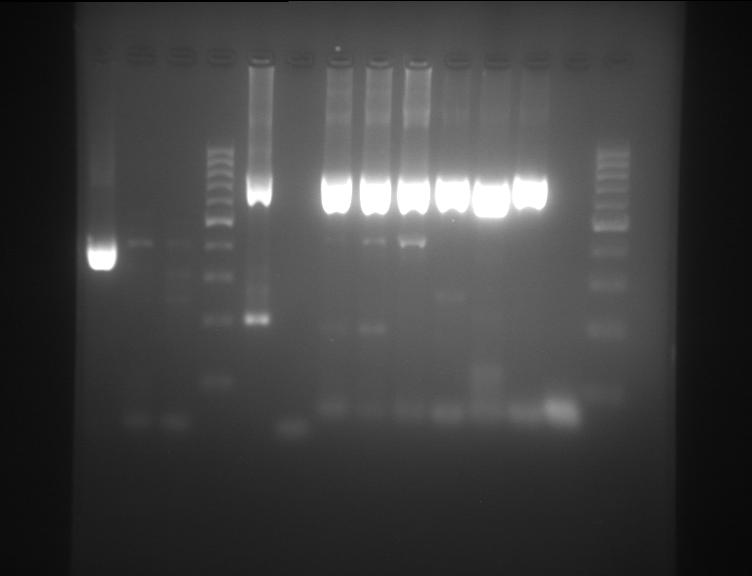


**Fig. S2 Original**
